# Diaphragm dysfunction after severe COVID-19: An ultrasound study

**DOI:** 10.3389/fmed.2022.949281

**Published:** 2022-08-24

**Authors:** Alain Boussuges, Paul Habert, Guillaume Chaumet, Rawah Rouibah, Lea Delorme, Amelie Menard, Matthieu Million, Axel Bartoli, Eric Guedj, Marion Gouitaa, Laurent Zieleskiewicz, Julie Finance, Benjamin Coiffard, Stephane Delliaux, Fabienne Brégeon

**Affiliations:** ^1^Faculté de Médecine, Center for Cardiovascular and Nutrition Research, C2VN, INSERM 1263, INRAE 1260, Aix-Marseille University, Marseille, France; ^2^Explorations Fonctionnelles Respiratoires, Hôpital Nord, APHM, Marseille, France; ^3^Département d’Imagerie, Hôpital Nord, APHM, LIIE, Aix-Marseille University, Marseille, France; ^4^ALTRABIO, Lyon, France; ^5^IRD, IHU-Méditerranée Infection, Marseille, France; ^6^Unité Post COVID, Service de Médecine Interne, Hôpital Nord, APHM, Marseille, France; ^7^Microbes Evolution Phylogeny and Infections (MEPHI), IHU-Méditerranée Infection, APHM, Aix-Marseille University, Marseille, France; ^8^Département de Radiologie, CNRS, CRMBM, Hôpital Timone, APHM, Aix-Marseille University, Marseille, France; ^9^Department of Nuclear Medicine, CNRS, Centrale Marseille, Institut Fresnel, Hôpital Timone, CERIMED, APHM, Aix-Marseille University, Marseille, France; ^10^Clinique des Bronches, Allergie et Sommeil, Hôpital Nord, APHM, Marseille, France; ^11^Service d’Anesthésie et Réanimation, Hôpital Nord, Marseille, France; ^12^Département des Maladies Respiratoire et Transplantation Pulmonaire, Hôpital Nord, APHM, Aix-Marseille University, Marseille, France

**Keywords:** chest ultrasonography, thickening fraction, SARS-CoV-2, diaphragm motion, respiratory physiotherapy

## Abstract

**Background:**

SARS-CoV-2 infection can impair diaphragm function at the acute phase but the frequency of diaphragm dysfunction after recovery from COVID-19 remains unknown.

**Materials and methods:**

This study was carried out on patients reporting persistent respiratory symptoms 3–4 months after severe COVID-19 pneumonia. The included patients were selected from a medical consultation designed to screen for recovery after acute infection. Respiratory function was assessed by a pulmonary function test, and diaphragm function was studied by ultrasonography.

**Results:**

In total, 132 patients (85M, 47W) were recruited from the medical consultation. During the acute phase of the infection, the severity of the clinical status led to ICU admission for 58 patients (44%). Diaphragm dysfunction (DD) was detected by ultrasonography in 13 patients, two of whom suffered from hemidiaphragm paralysis. Patients with DD had more frequently muscle pain complaints and had a higher frequency of prior cardiothoracic or upper abdominal surgery than patients with normal diaphragm function. Pulmonary function testing revealed a significant decrease in lung volumes and DLCO and the dyspnea scores (mMRC and Borg10 scores) were significantly increased in patients with DD. Improvement in respiratory function was recorded in seven out of nine patients assessed 6 months after the first ultrasound examination.

**Conclusion:**

Assessment of diaphragm function by ultrasonography after severe COVID-19 pneumonia revealed signs of dysfunction in 10% of our population. In some cases, ultrasound examination probably discovered an un-recognized pre-existing DD. COVID-19 nonetheless contributed to impairment of diaphragm function. Prolonged respiratory physiotherapy led to improvement in respiratory function in most patients.

**Clinical trial registration:**

[www.cnil.fr], identifier [#PADS20-207].

## Introduction

Acute respiratory failure is the most severe complication of COVID-19. Hypoxia occurs secondary to interstitial pneumonia and inflammatory lesions, leading to acute respiratory distress syndrome in some patients. Older age and various comorbidities such as cardiac and respiratory diseases, diabetes, and obesity have been associated with increased COVID-19 severity (1). Some patients require ventilatory support via facial mask or tracheal intubation. Mechanical ventilation can be extended to several days or even weeks. Early impairment of diaphragmatic function is thought to occur in intensive care unit (ICU) patients submitted to mechanical ventilation (2). Furthermore, after a long stay in ICU, various factors can contribute to the impairment of diaphragmatic function (2–4). The muscle wasting observed in some patients is a generalized phenomenon, described as ICU-acquired weakness. The respiratory muscle weakness results from various mechanisms, such as the impact of oxidative stress and decreased protein synthesis with or without increased protein degradation. Structural changes including fiber remodeling from slow to fast fibers are also involved in the impairment of diaphragmatic function.

Diaphragmatic function in COVID-19 patients can be impaired by several mechanisms. SARS-CoV-2 viral infiltration into the diaphragm of COVID-19–ICU patients has been reported based on pathological findings (5). Furthermore, increased expression of genes involved in fibrosis associated with histological evidence of fibrosis in the diaphragm muscle have been found in COVID-19 patients but were not observed in control-ICU patients (5). Lastly, neurological manifestations secondary to cerebral or peripheral nerve injuries have been observed in COVID-19 patients. Systemic inflammation and direct neuronal infection by the virus have been shown to be involved in these neurological lesions (6–8).

In some patients, after the acute phase of SARS-CoV-2 infection, persistent clinical impairments such as dyspnea and decreased physical capacity have been observed. Furthermore, hemidiaphragm paralysis has been reported after COVID-19 (9). Nevertheless, the contribution of diaphragmatic dysfunction (DD) to the impairment of respiratory function is currently unknown. The present study was, therefore, designed to assess the frequency and the risk factors for DD in patients recovering from COVID-19.

## Materials and methods

### Population included

This observational study was conducted in a French University Hospital (North Hospital, APHM, France). The study conformed to the general data protection regulation chart and was registered on the French health data registration portal under #PADS20-207. Patients with persistent clinical impairments after the acute infectious disease stage were selected from a medical consultation designed to assess the quality of recovery after COVID-19. To be included in our study, patients had to have suffered from severe COVID-19 pneumonia (the patients had been admitted to the hospital to receive supplemental oxygen or were submitted to mechanical ventilation). The diagnosis of COVID-19 pneumonia had to be supported by a clinical picture including respiratory difficulties associated with a radiologic pattern-compatible image of pneumonia and SARS-CoV-2 infection confirmed by PCR test. The medical consultation, pulmonary function testing (PFT), and diaphragm ultrasound were scheduled between 3 and 4 months after the hospital discharge for the acute phase of COVID-19 and were undertaken in the PFT lab of the North Hospital.

The medical consultation was designed to identify patients at risk of DD in the screened population. To detect patients at risk for DD, specific parameters were recorded from the patient’s medical history such as a history of trauma, surgery, and neurological, cardiac, or respiratory diseases. The clinical impairments experienced by the patients during the COVID-19 acute phase were also recorded. To assess the severity of the COVID-19, the need for ICU admission, mechanical ventilatory support, and the duration of the support were examined. A chest CT scan was performed at a date close to the medical consultation. The lesions reported by the CT scan and the radiological criteria of severity, graded according to the French Radiology Society guidelines,^1^ were recorded [absent, 0; minimal 1- (<10%); moderate, 2- (10–25%); extensive, 3- (25–50%); severe, 4- (50–75%); and critical, 5- (>75%)].

Lastly, the questionnaire and the clinical examination investigated the persistent clinical impairments such as dyspnea, cough, pain, decreased physical capacity, disorders suggesting a neurological condition, and any other medical issues. The dyspnea severity was assessed with the modified Medical Research Council (mMRC) and Borg10 scales.

### Pulmonary function test

The pulmonary function test (PFTs) included spirometry and body plethysmograph to measure the vital capacity (VC) and the total lung capacity (TLC) (PFT MasterLab Jaeger plethysmograph, Bunnik, Netherlands). The lung gas diffusion capacity of carbon monoxide (DLCO) was measured using the single-breath method with helium dilution (10). Absolute values were compared to the lower limit of normal (LLN), and the mean values were predicted by the CECA 93 equations (11). The lung functional impairment was screened according to the ATS/ERS definitions (12).

### Ultrasound study

The ultrasonographic examinations were carried out by two experienced investigators (AB and JF), both of whom had performed more than 500 ultrasound examinations of the diaphragm before the beginning of the study. The investigator performing the ultrasound was blinded to the results of the medical consultation and the PFT results. Diaphragmatic function was assessed as both the motion and the thickness of the two hemidiaphragms. The ultrasound examinations were performed using a commercially available ultrasound machine (Vivid S60N, GE Medical System, Milwaukee, WI, United States) equipped with a cardiac probe (3Sc probe) for the diaphragm excursion measurements and a linear vascular transducer (9L probe) for the diaphragm thickness measurements. The examinations were performed with the patients in a seated position.

### Assessment of diaphragm excursions

The excursions of both hemidiaphragms were measured using M-mode, as previously reported (13). Briefly, the probe was positioned on the subcostal or low intercostal area between anterior and posterior interaxillary lines to visualize the right and left hemidiaphragms. The selection of the best incidence was first determined using two-dimensional mode (B-mode). The line was positioned to reach the posterior part of each hemi-diaphragm before applying the M-mode. For a perpendicular approach, anatomical M-mode was used.

The diaphragmatic motion was assessed under three conditions: during quiet breathing at tidal volume, during voluntary sniffing, and during a deep inspiration at total lung capacity. After proper placement of the calipers, the inspiratory diaphragm excursions were measured. Measurements were averaged from at least three different respiratory cycles, except for deep breathing, for which we selected the maximum excursion among several recorded maneuvers.

### Assessment of diaphragm thickness

The right and left hemidiaphragms were visualized below the phrenico-costal sinus near the anterior or the mid-axillary line at the eighth or ninth intercostal space, where the diaphragm abuts the rib cage (zone of apposition) (14, 15). The thicknesses of both hemidiaphragms were measured directly from the frozen B-mode images as the distance from the pleural membrane to the peritoneal membrane, at the end of expiration and at the end of a deep inspiration.

The thickening fraction (TF) was calculated as the following ratio: the thickness at the end of deep inspiration – the thickness at the end of expiration divided by the thickness at the end of expiration.

### Diagnosis of diaphragm dysfunction

Diaphragm dysfunction (DD) was diagnosed based on previously published ultrasound criteria (16).

#### Hemidiaphragm paralysis

In patients suffering from hemidiaphragm paralysis, no motion or paradoxical excursion were observed during quiet breathing. A paradoxical motion was recorded during voluntary sniffing and sometimes at the beginning of deep inspiration (17). Furthermore, a finding of hemidiaphragm paralysis should be supported by evaluation of the thickening fraction. A lack of significant thickening (less than 20%) or thinning of the hemidiaphragm should be observed in such patients (18).

#### Diaphragm dysfunction without complete paralysis

The diagnosis of DD without complete paralysis was based on various ultrasound criteria. The excursions during deep inspiration should be lower than the lower limit of normal (LLN) according to the side and gender, based on recently published reference values (19). In contrast, no criteria of complete paralysis should be recorded by the ultrasound examination. Consequently, no paradoxical motion should be observed during the various maneuvers, and the inspiratory thickening should be greater than 20%. The value of the thickening fraction was used to assess the severity of the dysfunction (20).

According to the ultrasound findings, the patients were classified as:

1. Mild hemidiaphragm dysfunction when the excursion was slightly less than the LLN during deep inspiration (excursion > LLN – 1 cm) and a normal or slightly decreased (>40%) TF.
2. Severe hemidiaphragm dysfunction in patients with a marked decrease in hemidiaphragm excursion (<LLN – 1 cm) associated with a marked decrease in the TF (<40%).

### Follow-up

In patients suffering from DD, a follow-up including a medical consultation, PFTs, and ultrasound examination was scheduled 6 months after the first assessment.

### Statistical analysis

The characteristics of the patients suffering from DD (anthropometric data, severity of the acute episode of COVID-19, prior diseases and comorbidities, residual clinical impairments at the time of the medical consultation, and PFT results) were compared with the population with normal diaphragm function.

Numerical data were compared with Students’ *t*-test. When the data were not normally distributed, a Mann-Whitney test was used.

For qualitative data such as comorbidities and past medical histories, the comparison between groups (i.e., between patients with normal diaphragm function versus patients with DD) was performed using a chi-squared test. Yate’s correction was used when small numbers were involved.

We then searched for the factors associated with DD, using a logistic regression model. To select variables for the final multivariate model, the Boruta random forest method (21) was used for all the variables in the dataset. Variables labeled as confirmed by the Boruta algorithm were retained. Previously, on this dataset, missing data imputation was performed with the missForest non-parametric method (22). Variables with more than 25% missing data were removed.

After Boruta selection, a simple algorithm that computes the Variable Inflation Factor (VIF) was performed on the selected variables. When the maximum VIF value among the variables was greater than two, the corresponding variable was excluded. The process was then repeated until the VIF score of every variable was less than two. This step is crucial to limit the collinearity between the explanatory variables. A logistic model was computed using the selected variables (after Boruta and VIF selection), with the variable to be explained as “abnormal diaphragm function (yes/no)”.

Differences between groups were considered significant at *p* < 0.05.

## Results

### Patients

**FIGURE 1:**
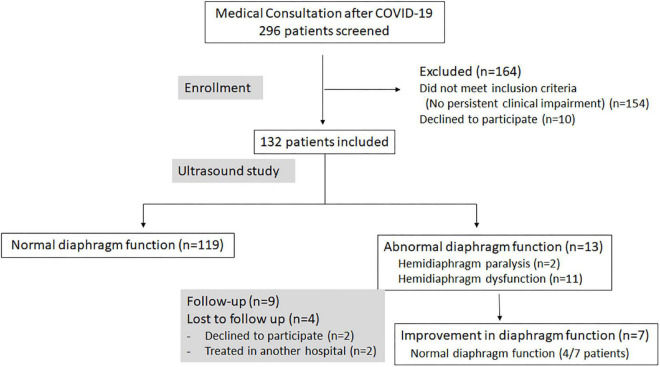
Flow diagram showing the enrollment, the results of ultrasound examinations, and the follow-up of patients participating in the study.

Of the 296 patients initially screened, 132 (85 men and 47 women) met the selection criteria and were included in the study (Figure 1). Their mean age was 56 ± 11 years, their mean height was 169 ± 9 cm, their mean weight was 80 ± 17 kg and their mean body mass index was 27.8 ± 5 kg/m^2^.

Forty-three (32%) were obese patients. The mean estimated weight loss induced by the SARS-CoV-2 infection as assessed at the study consultation was 4 kg.

The severity of the clinical status led to ICU admission for 58 patients (44%). Thirty-three patients required mechanical ventilation (25%), with a mean duration of 22 days.

Prior to their SARS-CoV-2 infection, 44 of the patients (33%) suffered from hypertension, 24 (18%) had other cardio-vascular diseases, 35 (26%) had diabetes, 18 (14%) had sleep apnea, and 11 (8%) had COPD.

Additionally, a history of chest trauma was found in three patients (2%), thoracic or cervical surgery in seven cases (5%), and abdominal surgery in 10 cases (8%).

Persistent respiratory difficulties were recorded in 103 patients (77%). The patients reported dyspnea in 87 cases (66%), cough in 32 cases (24%), and chest pain in 31 cases (23%). The other impairments were a decrease in physical capacity secondary to muscular weakness or pain in 34 cases (26%), dysesthesia in 42 cases (32%), cognitive disorders in 23 cases (17%), and palpitations in five cases (4%).

### Pulmonary function testing

Useable data were obtained from the PFTs in 92% of cases (122 out of 132). The results corresponded to normal function in 53 patients (43.5%), while a restrictive pattern was found in 35 patients (29%). The combination of low diffusion associated with a restrictive pattern was found in 14 cases (11.5%), an isolated low DLCO value was recorded in 15 cases (12%), and a mild obstructive pattern in five patients (4%).

### Ultrasound findings

Table 1 lists the results of the population studied.

**TABLE 1:** Measurements of hemidiaphragm excursions and thicknesses in the studied population.

	Women	Men	*P*-value
	
	Mean ± SD		
**Right hemidiaphragm**			
Quiet breathing (cm)	2 ± 0.4	2 ± 0.6	NS
Deep breathing (cm s^–1^)	4.6 ± 0.9	5.3 ± 1.4	<0.001
Expiratory thickness (mm)	1.8 ± 0.4	2.1 ± 0.4	<0.001
Inspiratory thickness (mm)	3.7 ± 0.9	4.2 ± 0.9	<0.01
Thickening fraction (%)	105 ± 46	97 ± 34	NS
**Left hemidiaphragm**			
Quiet breathing (cm)	2 ± 0.6	2.2 ± 0.6	NS
Deep breathing (cm)	4.5 ± 1.2	5.8 ± 1.3	<0.001
Expiratory thickness (mm)	1.7 ± 0.3	2 ± 0.4	<0.001
Inspiratory thickness (mm)	3.7 ± 0.9	4.1 ± 1	<0.05
Thickening fraction (%)	120 ± 50	109 ± 38	NS

The ultrasound examination detected 13 cases of abnormal diaphragm function, corresponding to hemidiaphragm paralysis in two patients and DD in 11 others, including six patients with mild dysfunction (four on one side and two on both sides) and five patients with severe dysfunction (3 cases on one side and 2 cases on both sides).

**FIGURE 2:**
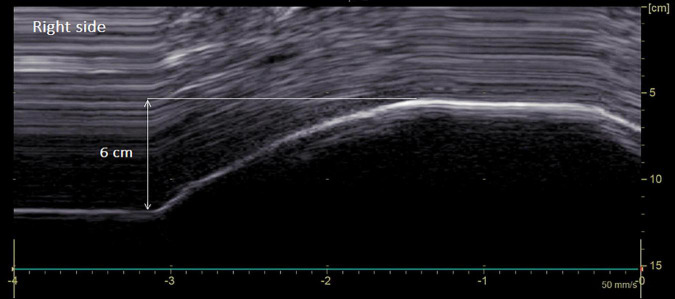
Diaphragmatic motion recorded by M-mode ultrasonography in a man suffering from left hemidiaphragm dysfunction. Hemidiaphragm excursions were measured by placing the first caliper at the foot of the inspiration slope on the diaphragmatic echoic line and by placing the second caliper at the apex of the curve (see arrow). *On the right side*: normal excursion during deep breathing (6 cm for a lower limit of normal = 4.1 cm).

**FIGURE 3:**
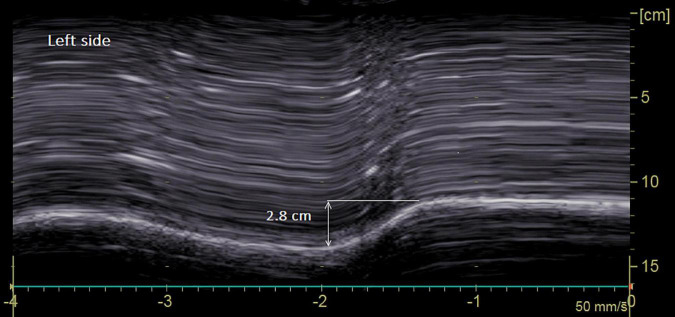
Diaphragmatic motion recorded by M-mode ultrasonography in a man suffering from left hemidiaphragm dysfunction. *On the left side*: marked decrease in hemidiaphragm excursion during deep breathing (2.8 cm for a lower limit of normal = 4.2 cm).

Figures 2, 3 illustrate the recording of diaphragmatic motion in a man suffering from left hemidiaphragm dysfunction.

The chest ultrasonography, the PFT results, and the medical history of the 13 patients with abnormal diaphragm function are presented in Table 2. In five of these 13 cases, risk factors for DD were recorded from the pre-existing medical history.

**TABLE 2:** Patients suffering from diaphragm dysfunction after SARS-CoV-2 infection: Ultrasound examination and pulmonary function testing (PFT).

Patient	Excursion (deep breathing)	TF	PFT	Patient history	Clinical picture	Follow-up (US)	PFT, clinical condition
1 M	Decrease in excursion of both hemidiaphragms (SHD)	<40% on both sides	Restrictive, low DLCO	Hypertension, sleep apnea	Cachexia	Slight improvement in DE	Restrictive, low DLCO, clinical improvement
2 W	Decrease in left hemidiaphragm excursion (MHD)	40% < TF < 60% on left side	Restrictive	**Scoliosis surgery**		Normalization	Normal PFT, normal clinical condition
3 M	Decrease in right hemidiaphragm excursion (MHD)	Nl	Restrictive, low DLCO	**Liver transplantation hypertension, diabetes**		Normalization	Restrictive, normal clinical condition
4 M	Decrease in left hemidiaphragm excursion (SHD)	TF < 40% on left side	Restrictive	**Cardiac surgery, heart failure, COPD**	Bilateral pleural effusion	–	No follow-up End-stage heart failure
5 W	Decrease in excursion of both hemidiaphragms (MHD)	Nl	Re strictive, low DLCO	**COPD**, **radiation trt for breast cancer**	Cachexia	–	No follow-up Declined to participate
6 M	Right hemidiaphragm paralysis	TF < 0 on right side	Obstructive	Obesity		Unchanged	Obstructive, normal clinical condition
7 M	Decrease in right hemidiaphragm excursion (SHD)	TF = 20% on right side	Restrictive	–	Thoracocentesis of right pleural effusion	Slight improvement	Restrictive, partial clinical improvement
8 M	Decrease in right hemidiaphragm excursion (SHD)	TF < 40% on right side	Restrictive	–		Improvement in DE, normalization TF	Restrictive, clinical improvement
9 W	Decrease in excursion of both hemidiaphragms (SHD)	TF < 40% on both sides	Restrictive	–	Myalgia, weakness, swallowing disorders, brain hypometabolism	Unchanged	Restrictive, severe clinical limitation, NIV support at home
10 M	Decrease in left hemidiaphragm excursion (MHD)	40% < TF < 60% on left side	Restrictive, low DLCO	**Lung transplantation**, **left pleural effusion**	Cachexia, allograft rejection	–	No follow-up 2*^nd^* lung transplantation
11 W	Decrease in excursion of both hemidiaphragms (MHD)	40% < TF < 60% on both sides	Low DLCO	–		Normalization excursions and TF	Low DLCO, normal clinical condition
12 M	Decrease in right hemidiaphragm excursion (MHD)	Nl	Restrictive	Hypothyroïdism	Guillain-Barré syndrome after COVID-19	-	No follow-up Declined to participate
13 M	Right hemidiaphragm paralysis	TF < 20% on right side	Restrictive, low DLCO	Obesity	**CT scan reporting diaphragm hernia**	Unchanged	Restrictive, no clinical improvement, surgery

M, male; W, female; TF, thickening fraction; PFT, pulmonary function test; US, ultrasound; DE, diaphragm excursion; DLCO, diffusive capacity for the lungs measured using carbon monoxide; COPD, chronic obstructive pulmonary disease; MHD, mild hemidiaphragm dysfunction; SHD, severe hemidiaphragm dysfunction; trt, treatment; NIV, non-invasive ventilation.

In bold: Recognized risk factors of diaphragm dysfunction recorded in the patient history or discovered by CT scan during COVID-19 (diaphragm hernia).

In one patient, it was possible to ascertain that the hemidiaphragm paralysis appeared after COVID-19, given the normal diaphragm position on the first chest X-ray upon hospital admission followed by abnormal elevation of the dome of the right hemidiaphragm on the post-discharge X-ray checkup. In the other patient, it was not possible to date the paralysis onset, but the CT scan performed at the acute phase of the infection revealed a diaphragm hernia, suggesting it probably arose prior to the COVID-19.

Pleural effusion was recorded in six patients. It was minimal and bilateral in two patients with DD, minor and on the left side in three patients with normal diaphragm function and in one patient with DD.

### Statistical analysis

Men had larger excursions at deep breathing than women, and their hemidiaphragms were thicker on both sides (Table 1).

Tables 3, 4 list the results of the comparison between patients suffering from hemidiaphragm dysfunction or paralysis (DD) versus patients with normal diaphragm function (ND) after COVID-19 severe pneumonia (univariate statistical analysis). The percentage of men was not significantly different between the groups (69% in the DD group vs. 64% in the ND group). The percentage of ICU admissions was not significantly different between the DD (54%) and the ND patients (43%). In the DD group, four patients out of 13 (31%) received mechanical ventilation compared to 29 out of 119 (24%) in the ND group (NS).

**TABLE 3:** Comparison between patients with diaphragm dysfunction or paralysis versus patients with normal diaphragm function after COVID-19 severe pneumonia.

Parameters	Patients		*P*-value
		
	Hemidiaphragm dysfunction or paralysis	Normal diaphragm function	
Number of patients	13	119	
Age (years)	57 ± 17	56 ± 10	NS
Weight (kg)	72 ± 19	81 ± 16	NS
Height (cm)	169 ± 8	169 ± 9	NS
BMI (kg^–1^ m^2^)	26 ± 6	28 ± 5	NS
**Comorbidity (in percentage)**			
Hypertension	46	32	NS
Cardiovascular disease	15	17	NS
Obesity	31	34	NS
Diabetes	15	28	NS
COPD	15	8	NS
Sleep apnea	8	14	NS
Cardiothoracic procedure or upper abdominal surgery	46	10	<0.001
**Clinical impairments (in percentage)**			
Dyspnea	85	64	NS
Chest pain	15	24	NS
Cough	31	24	NS
Amnestic disorders	31	16	NS
Myalgia	54	23	<0.05
Dysesthesia	31	32	NS

**TABLE 4:** Pulmonary function test.

Parameters	Patients		*P*-value
		
	Patients with hemidiaphragm dysfunction or paralysis	Patients with normal diaphragm	
Number of patients	13	119	
SVC (L)	2.4 ± 0.7	3.5 ± 0.9	0.001
SVC (% predicted)	63 ± 17	90 ± 17	<0.001
TLC (L)	4.3 ± 1	5.5 ± 1.2	<0.005
TLC (% predicted)	71 ± 14	90 ± 16	<0.001
DLCO	52 ± 23	71 ± 17	<0.05
Borg10 (median, 25–75%)	5 ± 3 (3–6)	3 ± 3 (2–5)	<0.05
mMRC (median, 25–75%)	3 ± 2 (1–3)	1 ± 2 (0–2)	<0.001

SVC, slow vital capacity; TLCO, total lung capacity; mMRC, Medical Research Council scale for dyspnea.

A chest CT scan could be performed on a date close to the medical consultation in 91% of the patients (120 out of 132 patients, 108 in the ND group, and 12 in the DD group).

The degree of lung parenchyma impairment as assessed by CT did not differ significantly between the groups (median 1 [1–2.25] in the DD group and 1 [0–2] in the ND group). The frequency of radiologic abnormal imaging was similar concerning ground-glass opacities (62 vs. 52%), consolidation (23 vs. 12%), and reticulation (31 vs. 29%) in the DD and the ND groups, respectively.

Patients with DD more frequently had a history of cardio-thoracic invasive procedures (including cardiac surgery, thoracic surgery, pleural effusion drainage, and atrial fibrillation ablation) or upper abdominal surgery (46% in total) than patients with normal diaphragm function (10%).

The results of the PFTs revealed significantly lower lung volumes and DLCO in patients with DD than in patients with normal diaphragm function. Furthermore, the dyspnea scores (mMRC and Borg10) were significantly increased in patients with DD.

Logistic regression analysis identified mMRC and TLC as the main factors associated with DD (Table 5).

**TABLE 5:** Results of the logistic regression analysis.

Predictor	Odds ratio	95% CI	Increment	*P*-value
Age (years)	1.005	0.952–1.064	1	0.86
BMI (kg^–1^ m^2^)	0.902	0.771–1.037	1	0.16
TLC (% predicted)	0.472	0.254–0.782	10	< 0.01
mMRC score	2.051	1.156–3.857	1	0.02

CI, confidence intervals; BMI, body mass index; TLC, total lung capacity; mMRC, Medical Research Council scale.

### Follow-up

In our population, all patients suffering from DD were managed by respiratory physiotherapy. A checkup visit could be performed for 9 out of these 13 patients. This consultation revealed significant improvement in diaphragm function in 7 out of 9 patients, at 6 months after the first ultrasound examination. This improvement led to normal diaphragm function in four patients, of whom two fully recovered normal PFT values.

## Discussion

This observational study performed on patients suffering from persistent adverse health effects 3–4 months after severe COVID-19 pneumonia found that their diaphragm function was nonetheless essentially normal. The mean values of excursions and thicknesses were close to the normal values previously reported in healthy volunteers assessed while in a seated position (19, 20). Furthermore, and as expected, men had larger excursions during deep breathing, and their hemidiaphragm thickness was increased compared to women.

Among the 132 patients, signs of DD were recorded by ultrasonography in 10% of cases (13 patients). Various conditions such as previous thoracic or abdominal surgery, thoracic or cervical trauma, prolonged ventilatory support, COPD, muscle wasting, neurological lesions, and myopathy are recognized as risk factors for DD (23). Nevertheless, such risk factors were not found consistently in the studied population.

The severity of the COVID-19 had made hospital admission for oxygen therapy necessary for all patients, with 44% being admitted to the ICU and 25% requiring mechanical ventilation. In our study, patients were assessed between 3 and 4 months after hospital discharge. Previous studies have shown that the impairment of diaphragm function induced by mechanical ventilation is a transitory phenomenon (24, 25) and that rehabilitation can improve DD (26). Here, the delay between the ICU discharge and the ultrasonography evaluation could explain why mechanical ventilation was not identified as an independent risk factor for DD.

The medical consultation carefully assessed whether there were risk factors for DD secondary to comorbidities or the medical/surgical history. By univariate analysis, prior surgical or invasive procedures (pleural effusion drainage, atrial fibrillation ablation) were more frequent in DD patients. Other recorded medical conditions that could contribute to DD, including broncho-pulmonary and cardiac disease, cachexia, and spinal surgery, are listed in Table 2. Consequently, in six patients, the ultrasound examination probably discovered an unknown and symptom-free DD induced by previous pathologies. Irrespective of whether a preexisting risk factor for DD was present in these patients, it is likely that the severity of the COVID-19 respiratory failure was promoted by the pre-existing DD through difficulties to increase the ventilatory regimen and protect against hypoxemia.

In seven patients, no risk factor was recorded in the medical history, so the impairment of diaphragm function could be attributed to the recent disease.

COVID-19 is thought to be able to induce impairment of diaphragm function through various mechanisms. An autopsy study has reported SARS-CoV-2 viral RNA located inside diaphragm myofibers in 15% of the patients who died while under mechanical ventilation (5). Impairment of skeletal muscle function has frequently been reported in both acute COVID-19 and post-acute sequelae of COVID-19. A pathogenesis resembling critical illness myopathy has been suggested, although the contribution of viral infiltration of the muscles and dysimmunity induced by SARS-CoV-2 infection are also recognized (27). Lastly, cases of myositis have been reported during COVID-19 recovery (28, 29). Lesions of the diaphragm muscle are, therefore, possible. In our population, the high percentage of patients experiencing muscle pain in the group with DD (54%) suggests persistent neuromuscular lesions several months after the acute phase of the SARS-CoV-2 infection.

SARS-CoV-2 can damage the peripheral and central nervous systems. Positron emission tomography (PET) imaging has revealed reduced metabolic activity in various regions of the brain (30, 31). Peripheral nervous system lesions have also been reported (32). It has been hypothesized that these neurological lesions may be due to inflammatory processes. In a pig model (33), oronasal inoculation of coronaviruses was associated with retrograde propagation of viruses into the medullary neurons of the brainstem. Direct brain invasion by SARS-CoV-2 is supported by human autopsy results. Indeed, viral particles were detected in neural and capillary endothelial cells in the frontal lobe of a COVID-19 patient (34).

In our population, COVID-19-induced neurological impairment could have also contributed to DD. One patient had brain hypometabolism and two patients had signs of peripheral nerve injury including one patient with Guillain-Barré syndrome occurring after the acute phase of the SARS-CoV-2 infection.

Lastly, another patient suffered from right hemidiaphragm paralysis secondary to phrenic nerve injury that arose during COVID-19. Indeed, this patient’s chest X-ray was normal at the beginning of the COVID-19 while he was hospitalized, whereas an elevation of the hemidiaphragm was found before discharge from the hospital. This complication has been reported previously (9, 35, 36). It appears to be rare during the acute phase, given that Abdeldayem et al. (37) detected an elevated hemidiaphragm in just 23 patients, out of 1,527 (1.5%) by CT scan. In the above-mentioned study, recovery was observed in 21 patients within 2 months. In the case we report here, no recovery occurred at 6 months of follow-up, although the clinical and PFT status nonetheless improved.

From the present work, statistical analysis of the data demonstrated that patients suffering from DD had a more severe respiratory status, including more severe dyspnea, a higher mMRC scale score, and significant impairment in PFT, including low TLC. Consequently, in patients reporting persistent respiratory difficulties after COVID-19, ultrasound examination can be performed to detect DD.

In our population, as also recommended by another team (38), the patients were managed by individually customized treatments that systematically included respiratory physiotherapy. In 9 patients out of 13 suffering from DD, ultrasound examination and PFT checkups were performed 6 months after the first assessment. Improvement in diaphragmatic function was noted in seven patients. Four patients reported no persisting clinical issues. It was observed that in patients with risk factors of DD before COVID-19, improvement in diaphragm function could be achieved by prolonged respiratory physiotherapy. This underscores the hypothesis of the contribution of the SARS-CoV-2 infection to impairment of diaphragmatic function even in patients with pre-existing risk factors of DD.

### Study limitations

As the detection of DD was infrequent in our study, the results of the statistical analysis should be interpreted with a degree of caution. Furthermore, in our population, the pathogenesis of DD most frequently remained hypothetical. Ultrasonography is an appropriate tool for detecting DD in combination with PFT. Nevertheless, to better understand the pathogenesis of the DD, it would be interesting to perform more extensive screening including assessment of phrenic nerve conduction and diaphragm muscle electromyography.

## Conclusion

Our study highlights the relevance of assessment of diaphragm function in patients suffering from persistent respiratory difficulties, with a high mMRC score and low TLC at PFTs, after COVID-19. Ultrasound examination can detect unknown pre-existing DD that can contribute to the severity of the initial picture and poor recovery. Furthermore, in some patients, the DD could be secondary to the SARS-CoV-2 infection through various mechanisms, including central or peripheral neurological lesions. In such patients, it is important to maintain respiratory physiotherapy for several weeks and to schedule systematic follow-ups to avoid complications.

## Data availability statement

The raw data supporting the conclusions of this article will be made available by the authors, without undue reservation.

## Ethics statement

The studies involving human participants were reviewed and approved in accordance with French legislation, Jardé law Article L1121-1 of December 31, 2016, the study fell within the legal framework of non-interventional research as performed on medical data collected during standard clinical care, and was not considered as study involving human beings. The research was reviewed and approved by the ethics board of the APHM institution (registered under number PADS20-207). The patients/participants provided their written informed consent to participate in this study.

## Author contributions

ABo and FB conceived and designed the study. PH, RR, LD, AM, MM, ABa, MG, LZ, BC, and SD assisted with the technical aspects of the protocol, recruited all the participants, and were involved in the acquisition of the data. ABo and JF performed the ultrasound examinations. ABa and GC analyzed the data and performed the statistical analysis. ABo, GC, and FB drafted the manuscript. PH and SD critically revised it for important intellectual content. All authors contributed to the article and approved the submitted version.

## Footnotes

1
http://www.sfrnet.org/sfr/

## References

[1] Gallo Marin B Aghagoli G Lavine K Yang L Siff EJ Chiang SS Predictors of COVID-19 severity: a literature review. *Rev Med Virol.* (2021) 31:1–10. 10.1002/rmv.2146 32845042 PMC7855377

[2] Jaber S Petrof BJ Jung B Chanques G Berthet JP Rabuel C Rapidly progressive diaphragmatic weakness and injury during mechanical ventilation in humans. *Am J Respir Crit Care Med.* (2011) 183:364–71. 10.1164/rccm.201004-0670OC 20813887

[3] Levine S Biswas C Dierov J Barsotti R Shrager JB Nguyen T Increased proteolysis, myosin depletion, and atrophic AKT-FOXO signaling in human diaphragm disuse. *Am J Respir Crit Care Med.* (2011) 183:483–90. 10.1164/rccm.200910-1487OC 20833824 PMC3056225

[4] Hussain SN Vassilakopoulos T. Ventilator-induced cachexia. *Am J Respir Crit Care Med.* (2002) 166:1307–8. 10.1164/rccm.2208004 12421738

[5] Shi Z de Vries HJ Vlaar APJ van der Hoeven J Boon RA Heunks LMA Diaphragm pathology in critically ill patients with COVID-19 and postmortem findings from 3 medical centers. *JAMA Intern Med.* (2021) 181:122–4. 10.1001/jamainternmed.2020.6278 33196760 PMC7670391

[6] Shehata GA Lord KC Grudzinski MG Elsayed M Abdelnaby R Elshabrawy HA. Neurological complications of COVID-19: underlying mechanisms and management. *Int J Mol Sci.* (2021) 22:4081. 10.3390/ijms22084081 33920904 PMC8071289

[7] Lyoo KS Kim HM Lee B Che YH Kim SJ Song D Direct neuronal infection of SARS-CoV-2 reveals cellular and molecular pathology of chemosensory impairment of COVID-19 patients. *Emerg Microbes Infect.* (2022) 11:406–11. 10.1080/22221751.2021.2024095 34962444 PMC8803065

[8] Swain O Romano SK Miryala R Tsai J Parikh V Umanah GKE. SARS-CoV-2 neuronal invasion and complications: potential mechanisms and therapeutic approaches. *J Neurosci.* (2021) 41:5338–49. 10.1523/JNEUROSCI.3188-20.2021 34162747 PMC8221594

[9] Dandawate N Humphreys C Gordan P Okin D. Diaphragmatic paralysis in COVID-19: a rare cause of postacute sequelae of COVID-19 dyspnoea. *BMJ Case Rep.* (2021) 14:e246668. 10.1136/bcr-2021-246668 34815229 PMC8611419

[10] Macintyre N Crapo RO Viegi G Johnson DC van der Grinten CP Brusasco V Standardisation of the single-breath determination of carbon monoxide uptake in the lung. *Eur Respir J.* (2005) 26:720–35. 10.1183/09031936.05.00034905 16204605

[11] Quanjer PH Tammeling GJ Cotes JE Pedersen OF Peslin R Yernault JC. Lung volumes and forced ventilatory flows. Report working party: standardization of lung function testing. *Eur Respir J.* (1993) 6:5–40. 24576915 10.1183/09041950.005s1693

[12] Pellegrino R Viegi G Brusasco V Crapo RO Burgos F Casaburi R Interpretative strategies for lung function tests. *Eur Respir J.* (2005) 26:948–68. 10.1183/09031936.05.00035205 16264058

[13] Boussuges A Gole Y Blanc P. Diaphragmatic motion studied by M-mode ultrasonography: methods, reproducibility, and normal values. *Chest.* (2009) 135:391–400. 10.1378/chest.08-1541 19017880

[14] Wait JL Nahormek PA Yost WT Rochester DP. Diaphragmatic thickness-lung volume relationship in vivo. *J Appl Physiol.* (1989) 67:1560–8. 10.1152/jappl.1989.67.4.1560 2676955

[15] Cohn D Benditt JO Eveloff S McCool FD. Diaphragm thickening during inspiration. *J Appl Physiol.* (1997) 83:291–6. 10.1152/jappl.1997.83.1.291 9216975

[16] Boussuges A Rives S Finance J Brégeon F. Assessment of diaphragmatic function by ultrasonography: current approach and perspectives. *World J Clin Cases.* (2020) 8:2408–24. 10.12998/wjcc.v8.i12.2408 32607319 PMC7322428

[17] Boussuges A Brégeon F Blanc P Gil JM Poirette L. Characteristics of the paralysed diaphragm studied by M-mode ultrasonography. *Clin Physiol Funct Imaging.* (2019) 39:143–9. 10.1111/cpf.12549 30325572

[18] Gottesman E McCool FD. Ultrasound evaluation of the paralyzed diaphragm. *Am J Respir Crit Care Med.* (1997) 155:1570–4. 10.1164/ajrccm.155.5.9154859 9154859

[19] Boussuges A Finance J Chaumet G Brégeon F. Diaphragmatic motion recorded by M-mode ultrasonography: limits of normality. *ERJ Open Res.* (2021) 7:00714–2020. 10.1183/23120541.00714-2020 33778044 PMC7983192

[20] Boussuges A Rives S Finance J Chaumet G Vallée N Risso JJ Ultrasound assessment of diaphragm thickness and thickening: reference values and limits of normality when in a seated position. *Front Med (Lausanne).* (2021) 8:742703. 10.3389/fmed.2021.742703 34778304 PMC8579005

[21] Kursa MB Rudnicki WR. Feature selection with the boruta package. *J Statist Softw.* (2010) 36:1–13.

[22] Stekhoven DJ Buehlmann P. ‘MissForest - nonparametric missing value imputation for mixed-type data’. *Bioinformatics.* (2012) 28:112–8. 10.1093/bioinformatics/btr597 22039212

[23] McCool FD Tzelepis GE. Dysfunction of the diaphragm. *N Engl J Med.* (2012) 366:932–42. 10.1056/NEJMra1007236 22397655

[24] Callahan LA Supinski GS. Rapid and complete recovery in ventilator-induced diaphragm weakness–problem solved? *J Appl Physiol.* (2013) 115:773–4. 10.1152/japplphysiol.00831.2013 23869069 PMC3764624

[25] Grassi A Ferlicca D Lupieri E Calcinati S Francesconi S Sala V Assisted mechanical ventilation promotes recovery of diaphragmatic thickness in critically ill patients: a prospective observational study. *Crit Care.* (2020) 24:85. 10.1186/s13054-020-2761-6 32164784 PMC7068963

[26] Dong Z Liu Y Gai Y Meng P Lin H Zhao Y Early rehabilitation relieves diaphragm dysfunction induced by prolonged mechanical ventilation: a randomised control study. *BMC Pulm Med.* (2021) 21:106. 10.1186/s12890-021-01461-2 33781259 PMC8006630

[27] Soares MN Eggelbusch M Naddaf E Gerrits KHL van der Schaaf M van den Borst B Skeletal muscle alterations in patients with acute Covid-19 and post-acute sequelae of Covid-19. *J Cachexia Sarcopenia Muscle.* (2022) 13:11–22. 10.1002/jcsm.12896 34997689 PMC8818659

[28] Hannah JR Ali SS Nagra D Adas MA Buazon AD Galloway JB Skeletal muscles and Covid-19: a systematic review of rhabdomyolysis and myositis in SARS-CoV-2 infection. *Clin Exp Rheumatol.* (2022) 40:329–38. 10.55563/clinexprheumatol/mkfmxt 35225218

[29] Uslu S. Myositis due to COVID-19. *Postgrad Med J.* (2021) 97:399. 10.1136/postgradmedj-2021-139725 33589489 PMC7886659

[30] Guedj E Campion JY Dudouet P Kaphan E Bregeon F Tissot-Dupont H 18F-FDG brain PET hypometabolism in patients with long COVID. *Eur J Nucl Med Mol Imaging.* (2021) 48:2823–33. 10.1007/s00259-021-05215-4 33501506 PMC7837643

[31] Rudroff T Workman CD Boles Ponto LL. 18F-FDG-PET imaging for post-COVID-19 brain and skeletal muscle alterations. *Viruses.* (2021) 13:2283. 10.3390/v13112283 34835088 PMC8625263

[32] Abu-Rumeileh S Abdelhak A Foschi M Tumani H Otto M. Guillain-Barré syndrome spectrum associated with COVID-19: an up-to-date systematic review of 73 cases. *J Neurol.* (2021) 268:1133–70. 10.1007/s00415-020-10124-x 32840686 PMC7445716

[33] Andries K Pensaert MB. Immunofluorescence studies on the pathogenesis of hemagglutinating encephalomyelitis virus infection in pigs after oronasal inoculation. *Am J Vet Res.* (1980) 41:1372–8. 6255837

[34] Paniz-Mondolfi A Bryce C Grimes Z Gordon RE Reidy J Lednicky J Central nervous system involvement by severe acute respiratory syndrome coronavirus-2 (SARS-CoV-2). *J Med Virol.* (2020) 92:699–702. 10.1002/jmv.25915) 32314810 PMC7264598

[35] Maurier F Godbert B Perrin J. Respiratory distress in SARS-CoV-2 without lung damage: phrenic paralysis should be considered in COVID-19 infection. *Eur J Case Rep Intern Med.* (2020) 7:001728. 10.12890/2020_001728 32523929 PMC7279902

[36] Law SM Scott K Alkarn A Mahjoub A Mallik AK Roditi G COVID-19 associated phrenic nerve mononeuritis: a case series. *Thorax.* (2022) 77:834–8. 10.1136/thoraxjnl-2021-218257 35459747

[37] Abdeldayem HE Abdelrahman AS Mansour MG. Recognition of phrenic paralysis as atypical presentation during CT chest examination of COVID-19 infection and its correlation with CT severity scoring: a local experience during pandemic era. *Egypt J Radiol Nucl Med.* (2021) 52:156. 10.1186/s43055-021-00527-9

[38] da Costa KV de Souza ITC dos Santos Felix JV Furtado Brandao BF De Souza Fernandes VM Lugon Favero AB Efficacy of a rehabilitation protocol on pulmonary and respiratory muscle function and ultrasound evaluation of diaphragm and quadriceps femoris in patients with post-COVID-19 syndrome: a series of cases. *Monaldi Arch Chest.* (2022) 20:1–13. 10.4081/monaldi.2022.2206 35723642

